# A Comparison of Subarachnoid Block Characteristics Following Co-administration of Fentanyl Premixed With Hyperbaric Bupivacaine Versus Antecedent or Succedent to Hyperbaric Bupivacaine: A Randomized Controlled Study

**DOI:** 10.7759/cureus.63666

**Published:** 2024-07-02

**Authors:** Lipika Saxena, Avnish Bharadwaj, Kalpana Verma, Pooja Mongia, Gautam Lunia

**Affiliations:** 1 Anesthesiology, Mahatma Gandhi Medical College and Research Institute, Jaipur, IND; 2 Community Medicine, Sarder Patel Medical College, Bikaner, IND

**Keywords:** compare, randomized controlled study, hyperbaric bupivacaine, fentanyl, subarachnoid block

## Abstract

Background

Adjuvants are often used during subarachnoid block to enhance and prolong the analgesia and decrease the adverse effects of high doses of local anesthetic agents. Intrathecal fentanyl premixed with hyperbaric bupivacaine has been used in spinal anesthesia and compared with the sequential use of these drugs in separate syringes. However, given the paucity of literature, we conducted this study where premixed antecedent and succedent administration of intrathecal fentanyl with hyperbaric bupivacaine were compared in terms of flow dynamics, block characteristics, and hemodynamic alterations.

Methodology

This prospective, randomized, triple-blinded comparative study was conducted among 160 patients who were randomly allocated into four groups. Group A (n = 40) (control) received 3.0 mL (15 mg) of 0.5% hyperbaric bupivacaine and 0.5 mL of normal saline via a 5.0 mL syringe. Group B (n = 40) received 3.0 mL (15 mg) of 0.5% hyperbaric bupivacaine and 0.5 mL (25 µg) of fentanyl premixed via a single 5.0 mL syringe. Group C (n = 40) received 0.5 mL (25 µg) of fentanyl via a 1.0 mL syringe followed by 3.0 mL (15 mg) of 0.5% hyperbaric bupivacaine via a 5.0 mL syringe. Group D (n = 40) received 3.0 mL (15 mg) of 0.5% hyperbaric bupivacaine via a 5.0 mL syringe followed by 0.5 mL (25 µg) of fentanyl via a 1.0 mL syringe. The onset and regression of sensory and motor blockade, hemodynamic parameters, time to first rescue analgesia, and adverse events were observed. Data analysis was done using SPSS version 17.0 (SPSS Statistics Inc., Chicago, IL, USA).

Results

The mean time taken for the onset of sensory and motor blockade was least in Group D followed by Group C. Duration of sensory and motor blockade was prolonged in Group D. Patients in Group A experienced more hypotension than Groups B, C, and D. Requirement of rescue analgesia was delayed in Groups C and D.

Conclusions

Administering 25 µg (0.5 mL) of Fentanyl separately after 15 mg (3.0 mL) of 0.5% hyperbaric bupivacaine results in early onset and prolonged duration of sensory and motor blockade, intraoperative hemodynamic stability, the delayed requirement of rescue analgesia postoperatively, and fewer side effects compared to its co-administration as a premixed solution or antecedent to hyperbaric bupivacaine.

## Introduction

Hyperbaric bupivacaine is widely used in spinal anesthesia because it provides good neural blockade and a longer duration of action compared to other local anesthetics [1]. In addition to the dose-sparing effect, various neuraxial adjuvants are used along with local anesthetics to speed up the onset of neural blockade and reduce their side effects [2].

Intrathecal fentanyl is the opioid of choice as a neuraxial adjuvant. It is highly lipid soluble and has a rapid onset and short duration of action intrathecally due to re-distribution. It also enhances block quality by reducing visceral and somatic pain, thereby reducing postoperative pain scores and the need for postoperative analgesics [3].

Intrathecal drug distribution is influenced by mixing opioids and hyperbaric bupivacaine based on baracity [3]. Baricity is the ratio of the density of local anesthetic and cerebrospinal fluid (CSF). The density of CSF at 37°C is 1.00059, fentanyl is 0.99410 g/mL, and hyperbaric bupivacaine is 1.02360 g/mL. After adding fentanyl with a local anesthetic in the same syringe, the density of the solution becomes 1.01850 g/mL [4]. Hence, although the density of the hyperbaric solution reduces, it remains hyperbaric [4]. Varying the specific gravity of the solution to the order of 0.0006 can modify the distribution of local anesthetics intrathecally. Hyperbaric solutions disperse immensely in the direction of gravity, thus causing less variable blockade among patients [5].

We propose that CSF dynamics and pharmacokinetics may be affected differently when fentanyl is co-administered premixed with hyperbaric bupivacaine compared to when administered separately before or after it. Although intrathecal fentanyl premixed with hyperbaric bupivacaine has been used in spinal anesthesia and compared with the sequential use of these drugs via separate syringes, there is a paucity of literature with clear data mentioning which drug should be given first in the sequential co-administration.

Given this, our study might be the earliest to investigate the flow dynamics, clinical effects, and hemodynamic alterations after the co-administration of fentanyl with hyperbaric bupivacaine either via a single syringe or before and after it via a separate syringe. Unlike previous studies, we included a control group where no adjuvant was added to hyperbaric bupivacaine. We compared the differences in the onset and duration of sensory and motor blockade, time to first rescue analgesia, and incidence of adverse events when fentanyl is co-administered with hyperbaric bupivacaine in the same syringe or antecedent or succedent to it via separate syringes.

## Materials and methods

This prospective, randomized, triple-blinded, comparative study was conducted at Mahatma Gandhi Medical College and Hospital, Jaipur, between January 2020 to January 2021 after receiving approval from the institutional ethical committee. Triple blinding was ensured by hiding the group allocation, the name of the drug, and the technique of administration from the patient, the person who administered it, and the person who recorded the data. This study was registered in the Clinical Trial Registry of India (CTRI/2022/03/041442). A total of 160 patients were enrolled in this study after fulfilling the inclusion and exclusion criteria. Patients of either sex aged 18-60 years with the American Society of Anesthesiologists (ASA) Class I/II/III who were scheduled for elective lower limb orthopedic surgeries under subarachnoid block were included. Patients belonging to ASA Class IV and V and who were allergic to the study drugs were excluded from the study. After obtaining written informed consent, a thorough preoperative assessment of the patients posted for lower limb surgeries under spinal anesthesia was performed by an anesthesiologist a day before the surgery.

Sample size

The estimated sample size was based on sensory onset (T10) (minutes) among groups. For the sample size calculation, we defined a mean difference of 1.7 with 2.3 SD. We calculated the sample size at a 95% confidence interval, 80% power, and alpha level of 0.05 using the following formula: N = 2 * ((z_α/2_+ z_β_)^2^ ÷ (*ᵟ*_0_)^2^) × sd^2^. Hence, the sample size was calculated to be 40 in each group. The sample size calculation was done based on a previous study done by Malhotra et al. [1].

Study grouping

On arrival, patients were randomly allocated into the following four groups of 40 patients each using a lottery method: (1) Group A (n = 40) (control): 3.0 mL (15 mg) of 0.5% hyperbaric bupivacaine and 0.5 mL normal saline in a single 5.0 mL syringe. (2) Group B (n = 40): 3.0 mL (15 mg) of 0.5% hyperbaric bupivacaine and 0.5 mL (25 µg) of fentanyl premixed in a single 5.0 mL syringe. (3) Group C (n = 40): 0.5 mL (25 µg) of fentanyl in a 1.0 mL syringe followed by 3.0 mL (15 mg) of 0.5% hyperbaric bupivacaine in a 5.0 mL syringe. (4) Group D (n = 40): 3.0 mL (15 mg) of 0.5% hyperbaric bupivacaine in a 5.0 mL syringe followed by 0.5 mL (25 µg) of fentanyl in a 1.0 mL syringe.

After taking the patient to the operation theater, all baseline parameters were recorded, including electrocardiography, heart rate, non-invasive blood pressure, saturation, and respiratory rate.

Under all aseptic precautions, a subarachnoid block was given in L3-L4 or L4-L5 intervertebral space with a 25 G Quincke Spinal needle in the sitting position. After confirming the free flow of CSF, the drug was injected intrathecally according to the group allocated, keeping the bevel of the needle cephalad. Patients were immediately put in the supine position.

Surgery was allowed once the sensory blockade (T10 dermatome) and motor blockade (Modified Bromage Score 3) were achieved. The sensory block was checked using the pinprick method in a caudal to cephalad direction in the mid-axillary line every two minutes for 20 minutes, followed by every 10 minutes until the completion of surgery. Motor block was assessed using the Modified Bromage Scale.

Intraoperative vitals were measured just before and after the subarachnoid block, every 10 minutes for 30 minutes, and then half hourly till the end of the surgery. Rescue analgesia was given in the form of inj. tramadol 1.5 mg/kg IV when the Visual Analog Scale (VAS) score was ≥4. The total dose and time of analgesia required in the first 24 hours postoperatively were noted. Patients were also monitored for various intraoperative and postoperative complications.

Blinding

To ensure triple blinding, lottery chits were used which were opened by the designated consultant of the work area just before the administration of anesthesia. The drug was prepared using a sterile technique according to the group allocation. The drug was then handed over to the attending anesthesiologist who was unaware of the study design and the study groups. The main observer of the study was called after induction to collect data intraoperatively in the proforma and postoperatively for rescue analgesia. Hence, this study remained triple-blinded as the patient, the anesthesiologist administering the drug, and the observer collecting the data were all blinded.

Data analysis

Data analysis was done using SPSS version 17.0 (SPSS Statistics Inc., Chicago, IL, USA) Windows software program. Quantitative variables were expressed as mean ± SD and qualitative variables as percentages and proportions. The significance of differences in mean among the groups was inferred by the analysis of variance test and the significance of differences in proportion among the groups by the chi-square test. Statistical significance was assigned at p-values of less than 0.05.

## Results

All four groups were comparable concerning mean age, gender, ASA grading, and duration of surgery (Table 1).

**Table 1:** Demographic data. *: insignificant at p > 0.05); ^@^: chi-square test; ^#^: analysis of variance. Group A (control): 3.0 mL (15 mg) of 0.5% hyperbaric bupivacaine and 0.5 mL of normal saline via a 5.0 mL syringe. Group B: 3.0 mL (15 mg) of 0.5% hyperbaric bupivacaine and 0.5 mL (25 µg) of fentanyl premixed via a single 5.0 mL syringe. Group C: 0.5 mL (25 µg) of fentanyl via a 1.0 mL syringe followed by 3.0 mL (15 mg) of 0.5% hyperbaric bupivacaine via a 5.0 mL syringe. Group D: 3.0 mL (15 mg) of 0.5% hyperbaric bupivacaine via a 5.0 mL syringe followed by 0.5 mL (25 µg) of fentanyl via a 1.0 mL syringe. ASA = American Society of Anesthesiologists

Variables	Group A (control)	Group B	Group C	Group D	P-value
Age in years (mean ± SD)	41.00 ± 13.97	40.78 ± 15.53	39.83 ± 15.80	39.38 ± 15.15	1.000^#^
Gender					
Male (%)	31 (77.50)	32 (80.00)	31 (77.50)	30 (75.00)	1.000^@^
Female (%)	9 (22.50)	8 (20.00)	9 (22.50)	10 (25.00)	
ASA grade					
ASA grade I (%)	33 (82.50)	32 (80.00)	27 (67.50)	34 (85.00)	0.233^@^
ASA grade II (%)	7 (17.50)	7 (17.50)	9 (22.50)	5 (12.50)	
ASA grade III (%)	0 (0.00)	1 (2.50)	4 (10.00)	1 (2.50)	
Duration of surgery (mean ± SD) (minutes)	75.63 ± 28.13	75.38 ± 27.42	76.00 ± 32.79	75.88 ± 22.35	0.066^#^

Intragroup statistical analysis demonstrated that the onset of sensory blockade and motor blockade were significantly earliest in Group D and delayed in Group A (p < 0.001). The mean time taken for two-segment sensory regression and mean duration of motor blockade was significantly prolonged in Group D and brief in Group A (p < 0.001). The requirement of rescue analgesia was significantly delayed in Group D and earliest in Group A (p < 0.001) (Table 2).

**Table 2:** Intragroup analysis of block characteristics. Analysis of variance, significant at p < 0.05. Group A (control): 3.0 mL (15 mg) of 0.5% hyperbaric bupivacaine and 0.5 mL of normal saline via a 5.0 mL syringe. Group B: 3.0 mL (15 mg) of 0.5% hyperbaric bupivacaine and 0.5 mL (25 µg) of fentanyl premixed via a single 5.0 mL syringe. Group C: 0.5 mL (25 µg) of fentanyl via a 1.0 mL syringe followed by 3.0 mL (15 mg) of 0.5% hyperbaric bupivacaine via a 5.0 mL syringe. Group D: 3.0 mL (15 mg) of 0.5% hyperbaric bupivacaine via a 5.0 mL syringe followed by 0.5 mL (25 µg) of fentanyl via a 1.0 mL syringe.

Variables	Group A (control) (mean ± SD)	Group B (mean ± SD)	Group C (mean ± SD)	Group D (mean ± SD)	P-value
Onset of sensory block (T 10) (minutes)	3.28 ± 0.46	2.21 ± 0.24	1.95 ± 0.26	1.82 ± 0.34	<0.001
Time for two-segment regression (minutes)	52.98 ± 3.88	80.55 ± 4.45	97.15 ± 5.73	101.95 ± 6.03	<0.001
Onset of motor blockade (Modified Bromage 3) (minutes)	8.08 ± 1.58	5.72 ± 1.04	3.92 ± 0.74	2.92 ± 0.70	<0.001
Duration of motor blockade (Modified Bromage 0) (minutes)	174.73 ± 5.80	175.20 ± 13.03	228.00 ± 23.12	253.13 ± 24.04	<0.001
Time to first rescue analgesia (hours)	4.19 ± 0.53	7.80 ± 2.78	13.20 ± 5.83	13.28 ± 4.89	<0.001

Intergroup statistical analysis demonstrated that the mean time taken for the onset of sensory and motor blockade was significantly less in Group D compared to Group C, followed by Group B and Group A (D < C < B < A) (p < 0.05). The mean time taken for two-segment sensory regression was significantly more in Group D compared to Group C, followed by Group B and Group A (D > C > B > A) (p < 0.05) (Table 3).

**Table 3:** Intergroup analysis of block characteristics. Analysis of variance, significant at p < 0.05. Group A (control): 3.0 mL (15 mg) of 0.5% hyperbaric bupivacaine and 0.5 mL of normal saline via a 5.0 mL syringe. Group B: 3.0 mL (15 mg) of 0.5% hyperbaric bupivacaine and 0.5 mL (25 µg) of fentanyl premixed via a single 5.0 mL syringe. Group C: 0.5 mL (25 µg) of fentanyl via a 1.0 mL syringe followed by 3.0 mL (15 mg) of 0.5% hyperbaric bupivacaine via a 5.0 mL syringe. Group D: 3.0 mL (15 mg) of 0.5% hyperbaric bupivacaine via a 5.0 mL syringe followed by 0.5 mL (25 µg) of fentanyl via a 1.0 mL syringe.

		Onset of sensory blockade (T10)	Time for two-segment regression	Onset of motor blockade (Modified Bromage 3) (minutes)	Duration of motor blockade (Modified Bromage 0) (minutes)	Time to first rescue analgesia
		P-value	P-value	P-value	P-value	P-value
Group A	Group B	<0.001	<0.001	<0.001	0.833	<0.001
Group A	Group C	<0.001	<0.001	<0.001	<0.001	<0.001
Group A	Group D	<0.001	<0.001	<0.001	<0.001	<0.001
Group B	Group C	<0.001	<0.001	<0.001	<0.001	<0.001
Group B	Group C	<0.001	<0.001	<0.001	<0.001	<0.001
Group C	Group D	0.035	0.000	<0.001	<0.001	0.950

Duration of motor blockade was significantly prolonged in Group D compared to Group C, followed by Group B and Group A. However, Group A and Group B showed no significant difference (D > C < B = A) (p < 0.05) (Table 3).

The requirement of first rescue analgesia was significantly earliest in Group A compared to Group B, followed by Group C and Group D (p < 0.05). However, Group C and Group D showed no significant difference (p > 0.05) (Table 3).

**Figure 1:**
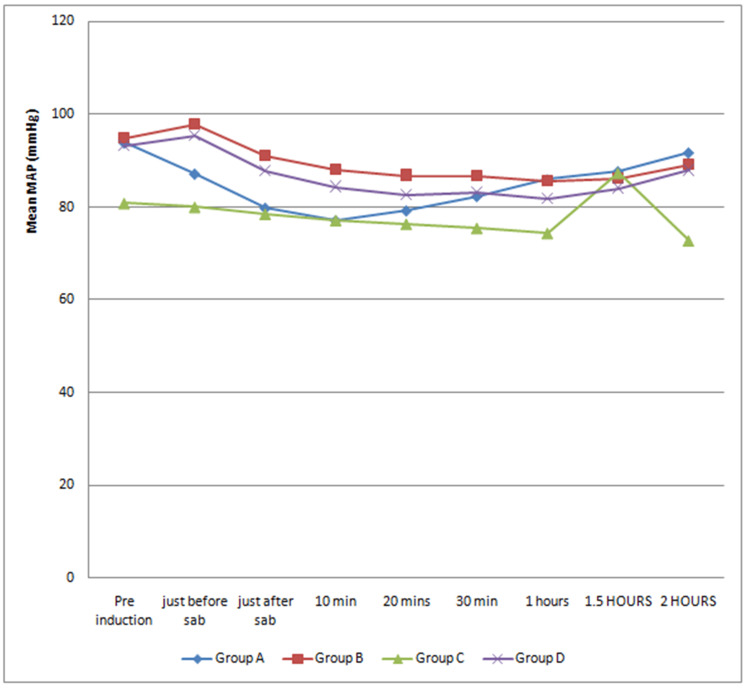
Trends in mean arterial pressure. Analysis of variance, significant at p < 0.05. Group A (control): 3.0 mL (15 mg) of 0.5% hyperbaric bupivacaine and 0.5 mL of normal saline via a 5.0 mL syringe. Group B: 3.0 mL (15 mg) of 0.5% hyperbaric bupivacaine and 0.5 mL (25 µg) of fentanyl premixed via a single 5.0 mL syringe. Group C: 0.5 mL (25 µg) of fentanyl via a 1.0 mL syringe followed by 3.0 mL (15 mg) of 0.5% hyperbaric bupivacaine via a 5.0 mL syringe. Group D: 3.0 mL (15 mg) of 0.5% hyperbaric bupivacaine via a 5.0 mL syringe followed by 0.5 mL (25 µg) of fentanyl via a 1.0 mL syringe.

There was a statistically significant decrease in mean arterial blood pressure up to a one-hour interval after giving subarachnoid block in Group A (control) compared to other Groups (p < 0.05) (Figure 1). Other vital parameters were comparable among all groups.

Hypotension occurred in nine (22.5%) patients in Group A (control), three (7.50%) patients in Group B, two (5%) patients in Group C, and one (2.50%) patient in Group D (p < 0.05). The incidence of other complications such as nausea, bradycardia, shivering, apnea, hypoventilation, itching, respiratory depression, and urinary retention was statistically insignificant among all groups (p > 0.05) (Table 4).

**Table 4:** Spinal anesthesia-related complications. *: significant at p < 0.05; chi-square test. Group A (control): 3.0 mL (15 mg) of 0.5% hyperbaric bupivacaine and 0.5 mL of normal saline via a 5.0 mL syringe. Group B: 3.0 mL (15 mg) of 0.5% hyperbaric bupivacaine and 0.5 mL (25 µg) of fentanyl premixed via a single 5.0 mL syringe. Group C: 0.5 mL (25 µg) of fentanyl via a 1.0 mL syringe followed by 3.0 mL (15 mg) of 0.5% hyperbaric bupivacaine via a 5.0 mL syringe. Group D: 3.0 mL (15 mg) of 0.5% hyperbaric bupivacaine via a 5.0 mL syringe followed by 0.5 mL (25 µg) of fentanyl via a 1.0 mL syringe.

	Group A control (n = 40)		Group B (n = 40)		Group C (n = 40)		Group D (n = 40)		P-value
	N	%	N	%	N	%	N	%	
Hypotension	9	22.50	3	7.50	2	5.00	1	2.50	0.012*
Bradycardia	4	10.00	0	0.00	0	0.00	0	0.00	0.008*
Shivering	6	15.00	2	5.00	2	5.00	2	5.00	0.306
Nausea	6	15.00	2	5.00	2	5.00	1	2.50	0.164

## Discussion

A subarachnoid block is a simple and advantageous technique compared to general anesthesia for lower limb surgeries. Such regional blockade is achieved with the help of various local anesthetic agents with or without adjuvants which have their own advantages and disadvantages. Hyperbaric bupivacaine is the most commonly used local anesthetic [6].

Various adjuvants administered concomitantly with local anesthetics have been shown to improve the quality of neural blockade and postoperative analgesia with varying degrees of success [7-10]. Fentanyl, a commonly used opioid, helps achieve successful spinal anesthesia even from subtherapeutic doses of local anesthetics. The clinical efficacy of fentanyl has been well documented to relieve visceral pain and enhance analgesia [11,12].

Ummenhofer et al. compared the pharmacokinetics of intrathecal opioids and found that fentanyl compared to sufentanyl spreads in the epidural space and gets cleared more rapidly due to its high volume of distribution [13]. Hence, the spread of hyperbaric bupivacaine in intrathecal space is affected by the co-administration of fentanyl as it alters the baricity of the solution and affects the sensory and motor block characteristics [14].

When fentanyl and bupivacaine are premixed, it dilutes the potency of fentanyl and decreases its supraspinal and spinal receptor occupancy thus producing less effect. On the other hand, when fentanyl is administered separately keeping the bevel of the needle cephalad, it occupies more receptors as it mixes freely with the CSF and spreads more cephalic. This is because the difference in the baricity of CSF and fentanyl is <0.006. Considering the spinal column as a closed space, we also speculate that bupivacaine, when given before fentanyl via a separate syringe, lies proximally in the subarachnoid space resulting in the early sensory blockade. The subsequent injection of highly lipophilic and low baricity fentanyl results in little agitation and generation of currents causing the cephalad spread of some amount of bupivacaine along with explaining early onset, longer duration of anesthesia and analgesia, and delayed regression of the blockade in the succedent group (Group D) compared to the antecedent group (Group C).

The motor blockade is less intense in premixed solution because fentanyl decreases the baricity of the solution leading to a greater scatter of drug molecules in the number of segments. However, when a hyperbaric drug is administered separately, the baricity of the drug remains unaltered. Hence, it settles in the lower part of the spinal canal and occupies a greater number of molecules in that particular segment providing dense and prolonged motor blockade, as seen in groups C and D. We also make a presumption that when bupivacaine is given before fentanyl (Group D), it starts its motor blockade early owing to its local anesthetic activity.

The premixed solution, being less viscous, spreads easily in the subarachnoid space and is less influenced by gravity. This causes immediate hemodynamic alterations such as hypotension that might require boluses of vasopressors. On the other hand, the hemodynamic profile remains more stable when a hyperbaric drug which is more viscous is given sequentially as its movement in intrathecal space is influenced by gravity. Further, we speculate that when bupivacaine is given before fentanyl (Group D) in the sequential blockade, delayed time from the sitting to the supine position helps in achieving compensatory mechanisms to prevent hemodynamic changes and hypotension.

In this study, the four groups were comparable concerning mean age, gender, ASA grading, duration of surgery, mean heart rate, mean arterial oxygen saturation, and mean respiratory rate.

The onset of sensory blockade was found to be significantly early in the succedent group on intra and intergroup comparison. These results were comparable to the studies by Keera et al. [15] and Kiruthika et al. [16].

The time taken for two-segment sensory regression was found to be significantly more in the succedent group on intragroup comparison. These results were comparable to the studies by Malhotra et al. [1], Bansal et al. [6], and Gaddam [17]. Intergroup comparison showed that the time taken for two-segment sensory regression was significantly more in the succedent group compared to the antecedent group. This result was contradictory to Malhotra et al. [1], who concluded that both these groups had insignificant differences.

The onset of motor blockade was also found to be early in the succedent group on inter and intragroup comparison. These results were comparable to the studies by Malhotra et al. [1] and Bansal et al. [6].

The duration of motor blockade was found to be prolonged in the succedent group on the intragroup comparison. These results were comparable to the studies by Malhotra et al. [1], Bansal et al. [6], and Desai et al. [18]. Intergroup comparison showed that the duration of motor blockade was significantly prolonged in the succedent group compared to the antecedent group. This result was contradictory to Malhotra et al. [1], who concluded that both these groups had insignificant differences.

There was a significantly delayed requirement of rescue analgesia in the succedent group, as observed in the intragroup comparison. Intergroup comparison showed that the requirement of first rescue analgesia was equally delayed in the antecedent and succedent groups. The results of our study were comparable to the studies by Malhotra et al. [1], Desai et al. [18], and Sachan et al [19].

Incidence of hypotension was significantly higher in the conventional hyperbaric bupivacaine (control) and premixed group compared to the antecedent and succedent groups, which was comparable to the studies by Malhotra et al. [1] and Keera et al. [15].

Postoperatively, six patients from Group A, two patients from Group B and Group C, and one patient from Group D complained of nausea which was statistically insignificant. The incidence of other complications between these groups was insignificant (p = 0.164) which was also comparable to the study by Malhotra et al. [1].

A few limitations in our study are worth mentioning. The study was conducted in patients undergoing lower limb orthopedic surgeries only. As the patients had undergone a variety of orthopedic procedures, there may be differences in the nature, type, and duration of postoperative pain attributing to the VAS score. Drug temperature, baricity, and injection rate were not measured to predict drug distribution relative to CSF.

## Conclusions

In patients undergoing lower extremities orthopedic surgeries under spinal anesthesia, administration of inj. fentanyl at a dose of 25 µg (0.5 mL) succedent to hyperbaric bupivacaine 0.5% (3.0 mL) results in early onset, prolonged duration of the motor as well as sensory blockade, intraoperative hemodynamic stability, delayed requirement of rescue analgesia postoperatively, and decreased incidence of side effects compared to conventional hyperbaric bupivacaine, or when co-administered as a premixed solution or antecedent to hyperbaric bupivacaine.
